# The hallmarks of a tradeoff in transcriptomes that balances stress and growth functions

**DOI:** 10.21203/rs.3.rs-2729651/v1

**Published:** 2023-04-12

**Authors:** Christopher Dalldorf, Kevin Rychel, Richard Szubin, Ying Hefner, Arjun Patel, Daniel C. Zielinski, Bernhard O. Palsson

**Affiliations:** 1 Department of Bioengineering, University of California, San Diego, La Jolla, USA; 2 Bioinformatics and Systems Biology Program, University of California, San Diego, La Jolla, USA; 3 Department of Pediatrics, University of California, San Diego, La Jolla, CA, USA; 4 Center for Microbiome Innovation, University of California San Diego, La Jolla, CA 92093, USA; 5 Novo Nordisk Foundation Center for Biosustainability, Technical University of Denmark, Kemitorvet, Building 220, 2800 Kongens, Lyngby, Denmark

## Abstract

Fit phenotypes are achieved through optimal transcriptomic allocation. Here, we performed a high-resolution, multi-scale study of the transcriptomic tradeoff between two key fitness phenotypes, stress response (fear) and growth (greed), in *Escherichia coli*. We introduced twelve RNA polymerase (RNAP) mutations commonly acquired during adaptive laboratory evolution (ALE) and found that single mutations resulted in large shifts in the fear vs. greed tradeoff, likely through destabilizing the *rpoB-rpoC* interface. RpoS and GAD regulons drive the fear response while ribosomal proteins and the ppGpp regulon underlie greed. Growth rate selection pressure during ALE results in endpoint strains that often have RNAP mutations, with synergistic mutations reflective of particular conditions. A phylogenetic analysis found the tradeoff in numerous bacteria species. The results suggest that the fear vs. greed tradeoff represents a general principle of transcriptome allocation in bacteria where small genetic changes can result in large phenotypic adaptations to growth conditions.

## Introduction

Maintaining optimal fitness in microorganisms requires navigating tradeoffs in resource allocation^1^. Expression profiling has shown that increasing ribosomal content (“greedy” genes) increases growth rates and additionally that decreasing the expression of stress response genes (“fearful” genes) is highly correlated with increasing growth rates in bacteria^1^. Thus, the fear vs. greed (f/g) tradeoff emerges as a key feature of transcriptome reallocation, in which cells that favor faster growth face the cost of diminished responsiveness to stresses^2^.

The f/g tradeoff was recently demonstrated as a property of the *E. coli* transcriptome through the use of a novel transcriptomic analysis method that defines sets of genes that are independently modulated, forming data-driven regulons termed iModulons^3^. The f/g tradeoff is characterized by the strong negative correlation between the activity levels of the RpoS (fear) and Translation (greed) iModulons. The f/g tradeoff involves an upregulation of ribosomal proteins that often are the limiting factor for increasing growth rate^4^ and a concurrent downregulation of stress-related genes. The tradeoff involves competition between the housekeeping and stress sigma factors (RpoD and RpoS), binding of ppGpp and DksA, and other regulatory mechanisms^5–7^. Many of these mechanisms directly involve RNA polymerase (RNAP).

Furthermore, the f/g tradeoff has been observed as an evolutionary adjustment in various studies^8–10^ and has been well documented in adaptive laboratory evolution (ALE) experiments^3,8,11,12^. In ALEs, strains are grown and propagated in the same condition for long periods of time, which creates a selection pressure that encourages endpoint strains to be enriched for mutations which increase growth rate. Therefore, ALE strains are expected to be as “greedy” as possible under the conditions used.

RNAP mutations have been shown to balance the f/g tradeoff and RNAP is one of the most common mutation targets during ALEs^13^. In a detailed study of two RNAP mutations found in the catalytic center, it was hypothesized these RNAP mutations adjust the tradeoff by destabilizing the *rpoB-rpoC* interface, thus affecting the binding of ppGpp to RNAP^2^. While many ALE mutations cluster in the catalytic center of RNAP, there are numerous other RNAP mutations found in ALE endpoint strains. These mutations can be found near regulator binding sites, regions known to be related to antibiotic resistance, important structural elements such as the flap domain and trigger loop, and in regions with no clear annotations^14–22^.

Previous to this study, the transcriptional impact of these RNAP mutations far from the catalytic core was unclear. Convergent RNAP mutations have been found in specific stress adaptation experiments^23–26^ often leading to the assumption that RNAP mutations reflect media adaptations, missing their underlying role in the f/g tradeoff. Here, we sought to expand our knowledge of the f/g tradeoff through a multi-scale study. We introduced twelve RNAP mutations and used computer simulations to infer how these mutations destabilize the *rpoB-rpoC* interface. We then obtained transcriptomes in various experimental conditions and used iModulon analysis to demonstrate that, despite structurally distinct locations, these mutations universally downregulate stress-related genes and upregulate growth-related genes in addition to some condition-specific adaptations. Finally, we compared the transcriptomes of various species to find that that f/g tradeoff is widely found across phylogeny. Thus, our multi-scale study elucidated key features of a central transcriptomic tradeoff between fear and greed and proposed that it is a general principle in microbiology.

## Results

### RNAP is a common mutational target in laboratory evolution

RNAP mutations are frequently fixed in ALEs, with 36% of evolved isolates in ALEdb, a database of mutations acquired during ALE^13^, containing at least one RNAP mutation: 6% have a *rpoA* mutation, 20% have a *rpoB* mutation, and 13% have a *rpoC* mutation. Twelve RNAP mutations were selected for experimental evaluation in this study using three primary criteria: (1) the frequency of occurrence of the mutation in ALE endpoints, (2) their structural location in relation to a known RNAP region of interest, such as effector binding sites, and (3) evidence of phenotypic impact of the mutation. The twelve chosen mutations and their characteristics are summarized in Supplemental Table 1. Each of these mutations were introduced into the genome of the model K-12 MG1655 strain of *E. coli* using CRISPR-based genome editing protocols (see Methods) to generate single mutation knock-in strains.

**Figure 2 –:**
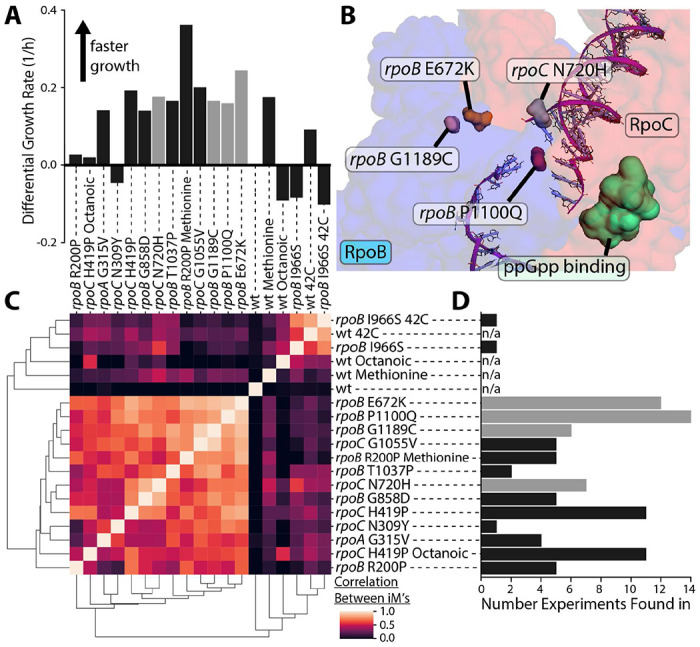
Transcriptome similarities and location of common ALE-acquired RNAP mutations. **(A)** The growth rates of the mutated strains relative to the wild-type control. **(B)** A subsection of RNAP (PDB 6OUL^27^) showing the location of common mutations with respect to the rpoB-rpoC interface and the ppGpp binding site, visualized using PyRosetta^28^. **(C)** Correlations between the activity levels of all iModulons between RNAP mutants under the same growth condition. The plot shows that all the twelve mutations have a similar impact on transcriptome composition. Mutations in the catalytic core have a near-identical impact on the transcriptome. **(D)** Number of laboratory evolution experiments that RNAP mutations are fixed in (number given is from a total of 743 ALE experiments found in ALEdb^13^). The gray bars in this panel and Panel C are the mutations grouped as “most common RNAP ALEdb mutations” in Figure 1 and are visualized on the RNAP structure in Panel B of this figure.

RNA-sequencing data was collected under aerobic growth on glucose M9 minimal media (see Methods). Some of the RNAP mutant strains were tested under specific stress conditions that were similar to the ALE experiment in which they were originally found (see Supplemental Table 2). All but one of the 12 mutants increased the growth rate and exhibited a shift toward greed in the f/g tradeoff in the transcriptome, as detailed below (Figure 2). The exception, *rpoB* I966S, arose during an evolution to high temperature growth^30^ and may therefore have had a stronger impact on temperature stability than regulation of expression.

### RNAP mutations destabilize the *rpoB-rpoC* interface and affect ligand binding

RNAP mutations have been shown to affect RNAP structurally in a variety of ways. Some of the most commonly found and widespread RNAP mutations are *rpoC* N720H, *rpoB* G1189C, *rpoB* P1100Q, and *rpoB* E672K (Figure 2). The physical mechanism for how these four mutations cause the tradeoff is not fully established, but some key properties are known. Structurally, they are all located near the *rpoB-rpoC* interface (*rpoB* E672K = 5.46 Å, *rpoB* P1100Q = 5.24 Å, *rpoB* G1189C = 8.97 Å, *rpoC* N720H = 10.09 Å). PyRosetta^28^ was used to calculate the impact of these mutations on the holoenzyme and found that all destabilized the *rpoB-rpoC* interface (*rpoB* E672K = −28.40 REU, *rpoB* P1100Q = −23.98 REU, *rpoB* G1189C = −5.26 REU, *rpoC* 1055V = −13.91 REU). This structural destabilization likely affects the ppGpp binding site and thus modifies its regulatory role^17^.

RNAP mutations modify RNAP’s interactions with sigma factors. Genes regulated by RpoS^31^, the general stress response sigma factor, showed on average a −0.33 change in log_2_ transcripts per million (tpm) expression when compared to the wild-type. The relatively small change (−0.059 change in log_2_ tpm) in genes regulated by RpoD, the housekeeping sigma factor, shows that these mutations differentially affect sigma factor functions (see Supplemental Table 3).

### RNAP mutations lead to upregulation of growth-related genes and downregulation of stress-related genes

The analysis of global changes in the transcriptome is difficult due to the high number of differentially expressed genes in many comparisons. Furthermore, comparing many conditions is challenging if pairwise differential expression of genes (DEG) plots are used^32^ (see Supplemental Figure 1). To overcome these challenges, we used the iModulon workflow^3,29^ to identify independently modulated gene sets (iModulons) and interpret their differential activity between all conditions used. This workflow uses independent component analysis (ICA) of a compendium (**X**) of RNA-sequencing data, which includes our samples of interest along with a variety of other experiments which help to separate source signals associated with transcriptional regulators^3,29^. The algorithm generates two output matrices: **M** (whose columns highlight the genes in each iModulon) and **A** (whose rows show the iModulon’s activity in every sample). Detailed information on each iModulon is available at iModulonDB.org^33^ and this study focuses primarily on the “*E. coli* PRECISE 2.0” dataset^29^; an *E. coli* database of RNA-sequencing data obtained under 422 growth conditions.

**Figure 3 –:**
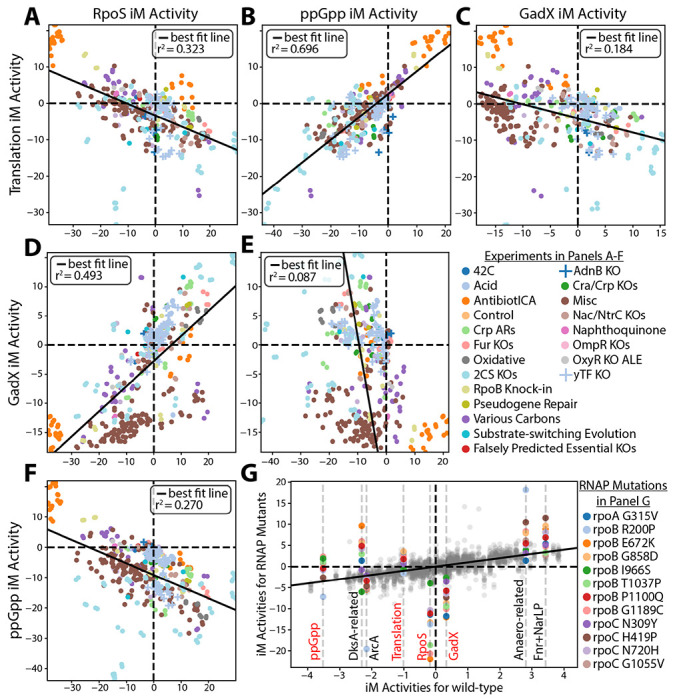
Reflections of the fear vs. greed tradeoff transcriptome in the relative activity levels of the translation and stress iModulons. **(A-F)** These plots show the relationship in activity levels between the greed (Translation and ppGpp) and fear (RpoS and GadX) iModulons. **(G)** The activity levels of various growth- and stress-related iModulons for the RNAP mutants, along with some other iModulons highly affected by said mutations. The gray dots are the activity levels of the other iModulons for all of the mutants. Red labeled iModulons are plotted in panels A-F.

Principal component analysis (PCA) of the iModulon activity matrix (**A**) shows that much of its variance and thus expression variation in general is explained by the RpoS and Translation iModulons’ activities (see Supplemental Table 4). The RpoS iModulon is the largest and the Translation iModulon is the fifth largest contributing factor to the highest variance explaining principal component (PC). GadX and ppGpp iModulons are also highly contributing factors to large variance explaining PC’s, adding additional dimensionality to f/g that is further explored in Figure 3. The f/g tradeoff is thus a major contributor to variation in the composition of the transcriptome.

The new RNA-sequencing data from the twelve new RNAP mutant strains was analyzed using ICA^3^. The iModulon activity levels in the new samples were compared to those in PRECISE 2.0. This database was used to compute the iModulons structure of the *E. coli* transcriptome^3^ and the gene composition of the key fear and greed iModulons is found in Supplemental Table 5. All of the four common ALEdb mutations strongly downregulate the activity of the RpoS iModulon and upregulate the activity of the Translation iModulon.

All of the twelve mutations introduced, except for *rpoB* I966S, have a large impact on the activity level of the RpoS iModulon similar to the two previously studied RNAP mutations^2^. The mutations in the catalytic center have the largest impact on RpoS iModulon activity levels, but mutations distant from this location can also strongly impact the activity of this iModulon. This suggests there is more complexity to the physical mechanism of this transcriptomic effect than the destabilization of the *rpoB-rpoC* interface. Both the frequency of occurrence and the effect of these mutations imply they are commonly fixed during growth rate selection (i.e., maximization of ‘greed’). Thus, a number of structurally similar mutations have a strong and similar effect on the composition of the transcriptome.

### Genome-scale models of proteome allocation quantitatively estimates the growth benefit of maximizing greed functions

While iModulons are an informative approach to reveal the hallmarks of changes in the expression state, they are not directly representative of the composition of the proteome. Creating iModulons from expression data requires the input RNA-sequencing data to be both centered to a control and normalized. This means the activity levels of iModulons for samples are entirely relative to each other and their magnitude range is constrained by the variance of the PRECISE dataset. We thus deployed a genome-scale model to reproduce the f/g tradeoff which allowed us to infer absolute measures of the proteome of cells undergoing said tradeoff. A genome-scale metabolism and expression (ME) model^34^ was run to maximize growth while constraining RpoS iModulon-associated reactions to a specified lower bound.

The resulting RpoS and Translation iModulons’ proteomic computed mass fractions were highly anti correlated (−0.9994) (see Supplemental Figure 2). A unit activity increase in the Translation iModulon has a 650% stronger effect on said iModulons’ genes’ proteome mass fraction than it does in the RpoS iModulon (see Methods). This implies that the small activity increases of the Translation iModulon seen in the f/g tradeoff and in the RNAP mutations may be having a larger effect than appears on the cell’s phenotype. This computational model also indicates that forced expression of the stress readiness genes reduce the expression of the growth promoting genes as experimentally observed.

### The fear vs. greed tradeoff additionally involves GAD and ppGpp iModulons

The f/g tradeoff was first visualized using the activity levels of the Translation and RpoS iModulons^3^. Since this study was published, the number of transcriptomes for *E. coli* has quadrupled^35^. The analysis of the larger data sets reveals additional dimensionalities to the f/g tradeoff. Several additional iModulon activity levels are correlated with growth rates, including the GadX and ppGpp iModulons. The former is related to acid stress and the latter to protein translation rates and the stringent response. GadX is highly correlated with RpoS (0.71) and negatively correlated with growth rates (−0.30) while ppGpp is strongly correlated with Translation (0.74) and has a weak positive correlation with growth rates (0.16). Correlation plots for each of these iModulon activity pairings are given in Figure 3A–F.

### Transcriptomic effects of RNAP mutations are also condition-specific adaptations

While the core group of common RNAP mutations downregulate stress-related iModulons and upregulate growth-related iModulons (Figure 3G), other RNAP mutations have more specific effects that are adaptations to the environments from which they were found. Supplemental Figure 4 shows two of these such mutations (*rpoB* R200P and *rpoA* G315V) from our set of twelve mutations.

The *RpoB* R200P mutation reflects a specific selection condition. It is found commonly in replicate methionine tolerance evolutions^25^ and it has two effects on the transcriptome: (1) during growth on methionine it activates the Translation iModulon and downregulates the RpoS iModulon to increase the growth rate compared to wild-type; and (2) during growth on M9 glucose it activates anaerobic response genes found in the Fnr-1, Fnr-2, and Anaero-related iModulons. Methionine contains sulfur and is thus a common target of reactive oxygen species (ROS) in *E. coli*^36^. The *RpoA G315V* mutation, which is further discussed in the Supplemental Information, adjusts the transcriptome similarly to a *crp* mutation^37^ and is commonly found in a *pgi* replacement evolution where it appears to be an adaptation to a failed replacement^24^.

Thus, there are RNAP mutations outside the core of the enzyme that reflect condition-specific effects on the transcriptome (see Supplemental Information for more cases). This observation leads to a wider examination of the effects of synergistic mutations with RNAP mutations that are selected under specific conditions.

### The genetic basis for the fear vs. greed tradeoff during ALE is condition-dependent

**Figure 4 -:**
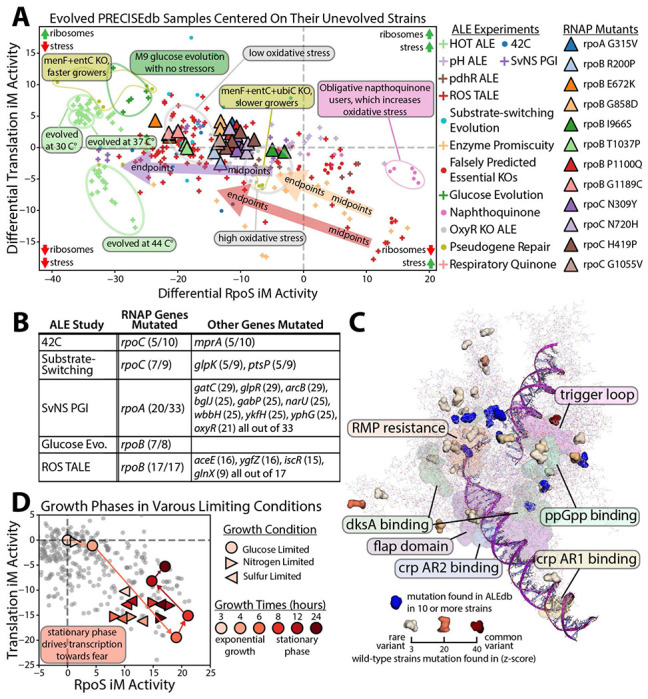
Fear vs. greed adaptations vary between laboratory and natural conditions. **(A)** The fear vs. greed iModulon activities of the laboratory evolved samples of PRECISE 2.0 centered on their respective unevolved wild-type strains’ iModulon activities. The triangles are the impact of the individual RNAP mutations from Figure 1. Specific annotations are given for different adaptation experiments showing variations in the effects of the selection pressure. Where midpoint strains are available, arrows indicate evolution from midpoint to endpoint strains. General effects of each quadrant are summarized in their respective corners. **(B)** Common endpoint strain mutations found in ALE experiments with both sequence and expression data available. The mutated genes are listed along with the number of evolved endpoint strains said gene is mutated in. Short explanations of each experiment are given in Supplemental Table 6. **(C)** The most common mutations found in ALEdb and the natural variants of RNAP (PDB 6OUL^27^) visualized using PyRosetta^28^. **(D)** The transition from growth to stationary phase, comes with down regulation of the Translation iModulon and upregulation of stress iModulons. This behavior shows clearly how growth rates go down and stress readiness increases, the opposite change to what happens during laboratory evolution to high growth rates. Data is from NCBI GEO GSE226643.

The fear and greed iModulons are correlated for both unevolved samples (−0.57 correlation) and evolved samples (−0.39 correlation, see Figure 4A), although evolved samples strongly favor greed. Different stressors lead to specific transcriptional adjustments along the f/g tradeoff to best favor growth as is annotated in Figure 4A.

In most laboratory evolutions with high stress conditions, evolution downregulates the RpoS iModulon over time. The cells initially use the RpoS iModulon to respond to nearly any stress, but eventually tune the stress response to the specific environment. In a reaction oxygen species experiment (labeled ROS TALE)^38^, initially the RpoS iModulon was highly active but as the cells evolved on paraquat most of the iModulon was downregulated while the expression of oxidative response genes in the iModulon were left largely unmodified (see Supplemental Figure 3). This transcriptional regulatory network adjustment, which was driven by convergent mutations, enabled the cells to grow faster in a ROS stress environment.

This evolutionary pull towards growth, and thus greed, in ALEs necessitates condition-specific convergent synergistic mutations (Figure 4B). These mutations are not strictly limited to RNAP. For example, *oxyR* is a common mutation target for evolution in oxidative stress^8^ and a *topA* mutation was a convergent target in a heat tolerance evolution^39^. This trend is widespread among ALEs, as 89% of experiments in ALEdb contain at least one gene that is mutated in 50% or more of their endpoint strains. If excluding RNAP genes, this drops to 80%. While RNAP mutations have the ability to favor growth across a wide variety of conditions, different genes are often better mutational targets for specific conditions.

### The fear vs. greed tradeoff is found in WT and across growth conditions

This ceaseless pull towards greed and away from stress readiness, however, is largely limited to laboratory conditions. The lack of overlap between the natural variants^40^ and the ALEdb mutations seen in Figure 4C implies that there are highly divergent evolutionary pressures on wild-type strains and their ALE counterparts.

It should be noted, however, that f/g changes are not limited to mutations acquired during evolution, as Figure 4D shows how the transcriptome composition falls on the tradeoff line in nutrient limited growth. Furthermore, when limiting nutrients drive the culture into the stationary phase, a time series of points shows how the transcriptome composition moves down the f/g tradeoff line. This movement shows how lower growth rates on entry into the stationary phase come with an increase in stress readiness. The f/g tradeoff is thus reflected in the various growth states of the WT strain as well as a transition in physiological states.

### The fear vs. greed tradeoff is found across the phylogenetic tree

**Figure 5 -:**
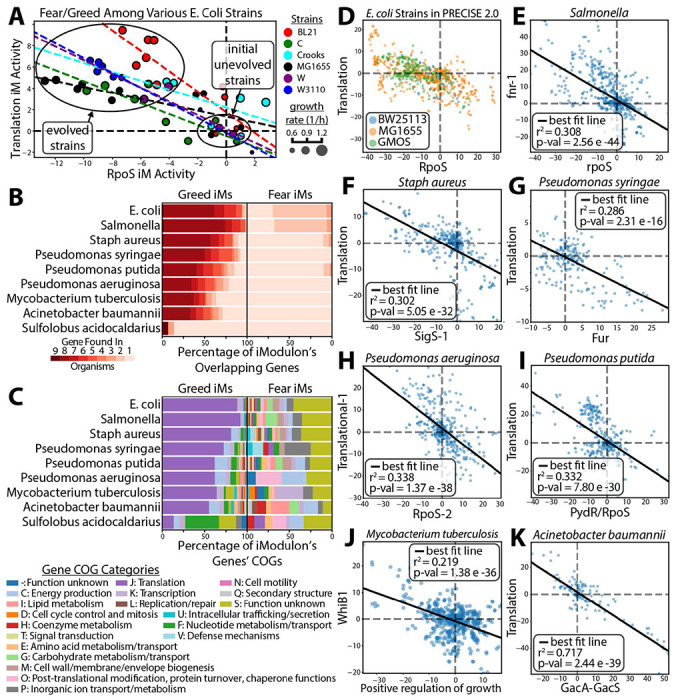
The fear vs. greed tradeoff is found across the phylogenetic tree. **(A)** The f/g tradeoff appears in ALEs across multiple E. coli strains^42^. **(B)** Percentage of genes found in common among translation and stress iModulons in different species. Many genes are commonly found in the Translation iModulons while the genes found in the fear iModulons are more disparate. Genes were matched to each other using Orthofinder on its default settings^43^. **(C)** The COG category of the genes of the greed and fear iModulons. **(D)** The f/g tradeoff among the E. coli strains found in PRECISE 2.0. GMOS are various genetically modified strains, typically gene knockouts. **(E) - (K)** The f/g tradeoff among a variety of species found in iModulonDB^33^. The p-value is calculated using a t-distribution Wald Test. The names of the iModulons are pulled from their respective data sets. Mycobacterium tuberculosis’ “Positive regulation of growth” iModuion mostly consists of stress-related antitoxin genes.

Finally, we searched the phylogenetic tree for other organisms exhibiting the f/g tradeoff. First we analyzed data from a multi-strain *E. coli* ALE study^41^. This analysis shows that the tradeoff was found in all the *E. coli* strains of the study (Figure 5A). Second, we examined iModulonDB^33^ for the presence of the f/g in other species (Figure 5D–K). The tradeoff was found in seven bacteria. Although the gene composition of the fear iModulons varies between species (likely a consequence of differing stresses in their natural environments), all of the primary greed iModulons consist of a highly similar set of ribosomal subunits and translational associated functions (Figure 5B–C). The presence of the f/g tradeoff across such a wide range of species implies that may be a global property of bacterial transcriptomes.^41^

## Discussion

We detail a general tradeoff in the bacterial transcriptome between growth rate and stress readiness. A major genetic component of this tradeoff lies in RNAP mutations, which affect the structure of RNAP and consequently the composition of the transcriptome. In RNAP mutants that arise from ALE studies, the modified transcriptome composition favors transcription of growth-related functions over stress-related functions. The tradeoff between fear and greed related functions was found across a wide range of wild-type bacterial strains. Interestingly, the fear vs. greed tradeoff has been described in many areas of science; such as economics^44^, game theory^45^, and psychology^46^. It has been elucidated here for microbiology through a multi-scale analysis.

A previous study compared two RNAP mutations^2^, *rpoB* E672K and *rpoB* E546V, and found that they destabilize the *rpoB-rpoC* interface^47^. Another study using *in vitro* assays linked an *rpoC* deletion from 3,611 to 3,619 bp to destabilizing the open complex of RNAP^48^. This destabilization came with decreased transcriptional pausing on the promoter, reduced RNAP’s open complex half-life, and increased elongation rates^48^. The impact of these mutations have been shown to be similar to strains with modified ribosomal operons, suggesting that these mutations are likely modifying ribosomal availability and/or distribution^9^. A recent study analyzing 45,000 ALE mutations and comparing them to wild-type mutations suggests that the wild-type mutations are under negative selection pressure, while ALE mutations are under positive selection pressure. This suggests that ALE mutations represent extreme mutations extenuating a preferred trait, thus amplifying the basis for the f/g tradeoff^40^.

The current study expanded upon current knowledge^2,48^ by analyzing the impact of twelve RNAP mutations to detail RNAP’s role as a global master regulator of the f/g tradeoff. The detailed molecular/structural mechanisms that underlie the tradeoff are not fully understood, but appear to involve destabilization of the *rpoB-rpoC* interface^2^, altered kinetic and regulatory properties^48^, and changes in the sigma factor use of RNAP.

The effects that RNAP mutations have on the transcriptome composition, however, are clear. The transcriptomic re-allocation involves a consistent set of iModulons with known functions. The relationship between the proteome and transcriptome functions enable genome-scale computational biology assessment of the phenotypic consequences of the reallocation^49^. Thus, a detailed understanding of the effects of the f/g tradeoff at the systems level has emerged. As the tradeoff involves resource allocations for improved fitness, it is important to contextualize particular RNAP mutations fixed in laboratory evolution studies and seek to identify epistatic mutations that are condition specific.

Finally, the phylogenetic distribution of the f/g tradoff is broad, suggesting that this tradeoff may emerge as a universal feature of the bacterial transcriptome. Thus, one can conjecture that this tradeoff was present in the last universal bacterial ancestor, and that RNAP may have played a role as a master transcriptional regulator in early life.

## Methods

### Creation of RNAP Mutations

RNAP mutations were created according to the protocol outlined in Zhao et al.^50^.

### RNA-sequencing

All samples were prepared and collected in biological duplicates. 3 ml of culture was added to 6 ml of Qiagen RNA-protect Bacteria Reagent after sample collection. This solution was then vortexed for 5 seconds, incubated at room temperature for 5 minutes, and then centrifuged. The supernatant was then removed and the cell pellet was stored at −80° C. The Zymo Research Quick-RNA MicroPrep Kit was used to extract RNA from the cell pellets per vendor protocol. On-columns DNase treatment was performed for 30 minutes at room temperature. Anti-rRNA DNA Oligo mix and Hybridase Thermostable RNase H^51^ was used to remove ribosomal RNA. Sequencing libraries were created using a Kapa Biosystems RNA HyperPrep per vendor protocol. RNA-sequencing reads were processed using https://github.com/avsastry/modulome-workflow. Data is available at NCBI GEO GSE227624.

### iModulon Computation

RNA-sequencing data was used to create iModulon activity levels of the mutated strains using PyModulon^3^ which is available at https://github.com/SBRG/pymodulon. Activities of iModulons were compared to samples from PRECISE 2.0^29^ which is easily accessible using iModulonDB^33^.

### Mutation Analysis

ALEdb^13^ was used for selecting the mutations for this study. Any *E. coli* strains on ALEdb were considered as potential sources for mutations. Mutations from the same sample but where one is from an isolate and one is from the population were considered to be just one instance of said mutation.

### Structural Analysis

Structural analysis was performed using PyRosetta^28^ using its default score function by mutating each residue in place and repacking the area within 10 Angstroms around it. The pdb files were downloaded from RCSB^52^. REU stands for Rosetta Energy Unit, which is PyRosetta’s unit for energy. For the holoenzyme mutation impacts, the pdb files used for the holoenzyme were 1L9U, 2A6E, 2CW0, 4YG2, and 4MEY, which were selected based on a review of bacterial RNAP^53^.

### Metabolic Model and Proteomic Calculations

The FoldME^34^ model was used for the metabolic modeling calculations. Supplemental Figure 2 was generated by iteratively increasing the lower bounds for the genes of the RpoS iModulon and recording the proteomic mass fraction of the Translation and RpoS iModulons’ genes until the model no longer ran. Proteome mass fraction to iModulon genes is the sum of the measured proteomic mass fractions of each enriched gene in an iModulon. This value is calculated for every sample and plotted against its corresponding PRECISE iModulon Activity. The proteomic calculations performed for this paper are well described in Patel et al^49^.

## Supplementary Material

1

## Figures and Tables

**Figure 1 -:**
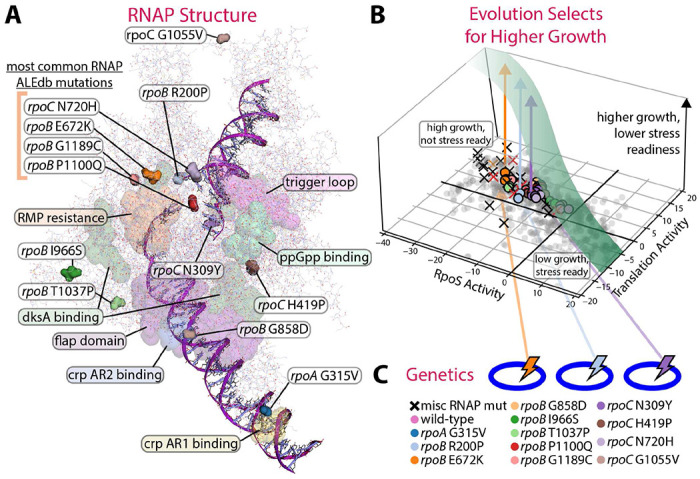
RNAP mutations alter the fear vs. greed tradeoff. **(A)** The structure of RNAP (PDB 6OUL^27^) is visualized using PyRosetta^28^, showing the location of mutations used in this study and highlighting some specific RNAP regions of interest^14–22^. The grouped mutations on the upper left are some of the most common mutations found in ALEdb^13^ and are further discussed in Figure 2. **(B)** Laboratory evolution leads to sequence variants which adjust the composition of the transcriptome leading to faster growth and repressed stress readiness. The f/g tradeoff on the transcriptome is shown (RpoS represents fear, Translation represents greed) along with the mutations’ qualitative impact on growth/stress readiness in the vertical axis visualized by the green curve. **(C)** The twelve mutations studied are listed, and the samples with the “misc RNAP mut” label are miscellaneous conditions in PRECISE 2.0^29^ with RNAP mutations^13,29^.
