# Supplementary material for: The hallmarks of a tradeoff in transcriptomes that balances stress and growth functions
Source: Res Sq. 2023 Apr 12:rs.3.rs-2729651. Preprint. [Version 1] doi: 10.21203/rs.3.rs-2729651/v1 (PMC10120744; doi:10.21203/rs.3.rs-2729651/v1)
Supplement: 1 [file NIHPPrs2729651v1-supplement-1.pdf]

Supplemental Information

Other RNAP mutations of special interested from our study

The *rpoA* G315V mutation affects the activities of Crp-1 and Crp-2 iModulons with the strongest impact on the maltose operons. This mutation was found in a *pgi* synthetic gene

replacement ALE[24] in nearly all strains that failed to integrate the exogenous *pgi* replacements. Presumably the loss of *pgi* required large changes to sugar import systems, thus necessitating this *rpoA* mutation to help downregulate maltose importers[54]. The mutation's effect on the Crp-1 iModulon is similar to one reported in a study that deactivated regions of *crp*[37] (see **Supplemental Figure 4E**). This similarity increases the likelihood that the mechanism of action for this *rpoA* mutation is to modify the *rpoA-crp* binding interface.

Some mutations predicted to have a specific stress response show no clear existence of one in our study. *RpoC* H419P was commonly found in octanoic acid tolerance studies and thus RNA-sequencing data was gathered for it both on M9 and on octanoic acid. Initially the *rpoC* H419P mutants had trouble growing on octanoic acid until the concentration was lowered, so perhaps an octanoic acid-specific adjustment would have been seen if a higher acid concentration was used. *RpoC* H419P has been previously introduced into *E. coli* and shown to be an adaptation to octanoic acid[26], so this issue is likely limited to our study.

RpoB I966S was commonly selected for in heat tolerance studies[39,55,56] and thus we chose to grow it in our study on high temperature conditions. These heat tolerance studies, however, were all carried out using *E. coli B-strains* and there was little effect of this mutation on the RNA-sequencing data so it appears this is a strain specific adaptation.

Supplemental Tables/Figures

Supplemental Table 1: The mutations created for this study and the reason for their inclusion.

**Gene**	**Mutation**	**Mutation Frequency (total strains found in)**	**Reason for Inclusion**
rpoA	G315V	12	Located nearby to crp AR1 binding site, found in pgi swap studies
rpoB	R200P	10	Found primarily in methionine tolerance experiments
rpoB	E672K	44	Very common mutation, located near the catalytic core of RNAP
rpoB	G858D	8	Proximity to rpoS binding site
rpoB	I966S	15	Found primarily in high temperature experiments
rpoB	T1037P	12	Mutation commonly found in drug-resistance experiments
rpoB	P1100Q	417	Most common mutation, located near the catalytic core of RNAP
rpoB	G1189C	28	Very common mutation, located near the catalytic core of

			RNAP
rpoC	N309Y	3	Found primarily in butanediol tolerance experiments
rpoC	H419P	26	Very common mutation found most often in octanoic tolerance experiments
rpoC	N720H	52	Very common mutation, located near the catalytic core of RNAP
rpoC	G1055V	8	Common mutation found far from any other mutations included

Supplemental Table 2: Experimental conditions where new RNA-sequencing profiles were generated.

Condition	Base Media	Glucose Concentration	Additional Supplements
Glucose	M9	4 g/L	none
Octanoic	M9	4 g/L	12 mM octanoic acid
Methionine	M9	4 g/L	30 g/L methionine
42C	M9	4 g/L	Run at 42° celsius

Supplemental Table 3: Sigma factors, their regulated genes, and the average expression change among the mutated strains created for this study.

Sigma Factor	Genes Regulated (according to RegulonDB[31])	Average Expression Change ($\log_2$ tpm)
Sigma 24 (*rpoE)*	*ahpF, apaG, apaH, bacA, bamA, bamB, bamC, bamD, bamE, bepA, bhsA, cca, clpX, degP, der, dnaE, dsbC, eptB, essD, fabZ, fadM, fkpA, ftnB, fusA, greA, gspA, gspB, hcp, hcr, hpf, ibaG, insK, lgoR, lhr, lon, lptA, lptB, lptD, lpxA, lpxB, lpxD, lpxP, lyxK, malQ, mscM, mzrA, narV, narW, opgG, opgH, pdxA, phoQ, plsB, prfB, psd, ptsN, rapZ, rclA, rclC, recJ, recR, rfaD, rnhB, rnlB, rpoD, rpoE, rpoH, rpoN, rrrD, rseA, rseB, rseC, rseD, rseP, rsmA, rutR, rzpD, sbmA, sgbE, sgbH, sgbU, sixA, skp, surA, tufA, uspD, waaC, waaF, waaL, wza, wzb, wzc, yaiW, ybaB, ybfG, ydhI, ydhJ, ydhK, yeaY, yfeK, yfeS, yfeX, yfeY, yfgD, yggN, yghF, ygiM, yhjJ, yiaK, yiaL, yiaM, yiaN, yiaO, yicI, yicJ, yidQ, yieE, yieF, yiiS, yoaC, yqjA, ytfJ*	-0.041
Sigma 28 (*rpoF*)	*aer, cheA, cheB, cheR, cheW, cheY, cheZ, flgK, flgL, flgM, flgN, fliA, fliC, fliD, fliE, fliF, fliG, fliH, fliI, fliJ, fliK, fliL, fliM, fliN, fliO, fliP, fliQ, fliR, fliS, fliT, fliZ, flxA, hemL, ispE, lolB, modA, modB, modC, motA,*	-0.053

	motB, oppA, oppB, oppC, oppD, oppF, pdeH, ppdA, ppdB, ppdC, prs, recC, tap, tar, tcyJ, trg, tsr, ves, yafW, yafX, ycgR, yecF, ygbK, ygdB, yhiL, yjcS, ykfB, ykfF, ykfG, ykfH, ykfI, ynjH	
Sigma 32 (*rpoH*)	*ackA, adiC, alaA, bssS, can, cas1, cas2, casD, casE, clpB, clpP, clpX, cnoX, cra, creA, creB, creC, crr, dnaJ, dnaK, dsbC, fkpB, ftsH, fxsA, gapA, glnS, groL, groS, grpE, hflC, hflK, hflX, hfq, holC, hslO, hslR, hslU, hslV, hspQ, htpG, htpX, ibpA, ibpB, ileS, ispH, lapA, lapB, ldhA, lipB, lnt, lon, lspA, mbiA, metA, mhpT, miaA, mlc, mngA, mngB, mpaA, mutL, mutM, narP, nfuA, nusB, osmF, pgpA, pgpC, phoP, phoQ, pncC, pphA, ppiD, prlC, ptsH, ptsI, pyrF, raiA, rapA, rdgB, recJ, rfaD, ribE, rlmE, rnlA, rpmE, rpoD, rsmJ, sdaA, slt, tadA, thiL, topA, trmA, tyrR, valS, waaC, waaF, waaL, xerD, yafD, yafE, yafU, ybeD, ybeX, ybeY, ybeZ, ybfE, yccE, yceI, yceJ, yciH, ycjF, ycjX, ycjY, ydeO, ydhQ, yeaD, yehR, yehW, yehX, yehY, yfbR, yfjV, yhdN, yiaA, yibA, yjaZ, yjhG, yjhH, yjhI, yjiT, ymjC, yrdA, yrfG, zntR*	-0.040
Sigma 38 (*rpoS*)	*aceE, aceF, acnA, acs, actP, ada, adhE, aidB, aldB, alkA, alkB, ansP, appA, appB, appC, appY, araF, araG, araH, ariR, artI, artM, artP, artQ, asr, astA, astB, astC, astD, astE, baeR, baeS, blc, blr, bolA, bsmA, btsT, btuF, cbpA, cbpM, cfa, cpxA, cpxR, crr, csgA, csgB, csgC, csgD, csgE, csgF, csgG, csiE, csrA, ddpA, ddpB, ddpC, ddpD, ddpF, ddpX, dgcC, dhaK, dhaL, dhaM, dhaR, dinB, dkgB, dmsD, dnaN, dps, ecnB, elaB, eno, envC, epd, evgA, evgS, fadL, fau, fbaA, fbaB, fic, folK, frdA, frdB, frdC, frdD, ftsA, ftsQ, ftsZ, fumC, gabD, gabP, gabT, gadA, gadB, gadC, gadE, gadW, gadX, galE, galK, galM, galT, gapA, glgA, glgC, glgP, glgS, glk, glsA, gltB, gltD, gltF, gor, gpmA, gpmM, gyrB, hchA, hdeA, hdeB, hdeD, hdfR, hmp, hofM, hofN, hofO, htrE, hyaA, hyaB, hyaC, hyaD, hyaE, hyaF, ihfA, ihfB, ilvY, iraD, katE, kbp, ldcC, lpd, lsrA, lsrB, lsrC, lsrD, lsrF, lsrG, luxS, mdtA, mdtB, mdtC, mdtD, mdtE, mdtF, metK, mglA, mglB, mglC, mlrA, mnmG, mpl, msyB, mtn, murP, murQ, murR, mutS, narU, nhaA, nhaR, ompF, ompN, osmB, osmC, osmE, osmF, osmY, otsA, otsB, oxyR, pabA, patA, patD, pcnB, pdeC, pdeR, pdhR, pfkA, pfkB, pgi, pgk, phoU, phr, poxB, pphA, ppk, ppx, pqiA, pqiB, pqiC, proP, proV, proW, proX, pstA, pstB, pstC, pstS, puuA, puuB, puuC, puuD, puuE, puuP, puuR, pykA, pykF, ravA, recF, rhaR, rhaS, rpoE, rpoH, rraA, rsd, rseA, rseB, rseC, rseD, rsmG, rssA, rssB, setA, sgrT, sodC, sohB, speB, speC, speG, sra, sucA, sucB, sucC, sucD, talA, tam, tktB, tolC, topA, tpiA, treA, treF, uspB, viaA, wrbA, xapA, xapB, xthA, yabI, yadS, yadV, yafN, yafO, yafP, ybaT, ybgA, ybiI, ybjP, yccJ, ycgZ, yciE, yciF, yciG, yciT, yciZ, ydbD, ydbK, ydcS, ydcT, ydcU, ydcV, yddG, ydhT, ydhU, ydhV, ydhW, ydhX, ydhY, yehW, yehX, yehY, yeiL, yffO, yffP, yggE, ygiB, ygiC, yhfG, yhiD, yhiM, yhjG, yiaG, yibQ, yihG, yjcH, ymgA, ymgC, ymjE, ynfB, ynfE, ynfF, ynfG, ynfH, ytfK, ytiC, ytiD, znuA*	-0.332
Sigma 54 (*rpoN*)	*acrD, actP, amtB, argT, aslB, astA, astB, astC, astD, astE, atoA, atoB, atoD, atoE, chaC, dcuD, ddpA, ddpB, ddpC, ddpD, ddpF, ddpX, emrD, fdhF, fhlA, flhC, flhD, focB, glnA, glnG, glnH, glnK, glnL, glnP, glnQ, glpQ, gltI, gltJ, gltK, gltL, gnsA, hisJ, hisM, hisP, hisQ, htpG, hycA, hycB, hycC, hycD, hycE, hycF, hycG, hycH, hycI, hydN, hyfA, hyfB, hyfC, hyfD, hyfE, hyfF, hyfG, hyfH, hyfI, hyfJ, hyfR, hypA, hypB, hypC,*	-0.057

	hypD, hypE, hypF, ibpB, nac, nikA, nikB, nikC, nikD, nikE, nikR, norV, norW, patA, potF, potG, potH, potI, prpB, prpC, prpD, prpE, pspA, pspB, pspC, pspD, pspE, pspG, puuP, radD, relA, rhaD, rpoE, rpoH, rseA, rseB, rseC, rseD, rtcA, rtcB, rtcR, rutA, rutB, rutC, rutD, rutE, rutF, rutG, ssnA, xapB, xdhA, xdhB, xdhC, yaaU, yahE, yeaG, yeaH, ygfK, yhdW, yhdX, yhdY, yhdZ, ymjE, zraP, zraR, zraS	
Sigma 70 (*rpoD*)	*accA, accB, accC, accD, aceA, aceB, aceE, aceF, aceK, acnA, acnB, acpP, acpS, acrA, acrB, acrD, acrE, acrF, acrR, acrZ, acs, actP, ada, add, adeD, adhE, adiA, agaA, agaC, agaD, agaI, agaR, agaS, agaV, agaW, ahpC, ahpF, aidB, alaC, alaE, aldA, alkA, alkB, allB, allC, allD, allE, alsA, alsB, alsC, alsE, alsR, amiA, amiB, ampC, ampG, amyA, ansB, appA, appB, appC, appY, apt, araA, araB, araC, araD, araE, araF, araG, araH, araJ, arcA, arfA, argA, argB, argC, argD, argE, argF, argG, argH, argI, argO, argR, argS, ariR, aroA, aroB, aroD, aroF, aroG, aroH, aroK, aroL, aroM, aroP, arsB, arsC, arsR, artI, artJ, artM, artP, artQ, ascB, ascF, asnA, asnC, aspA, asr, astA, astB, astC, astD, astE, atpA, atpB, atpC, atpD, atpE, atpF, atpG, atpH, atpI, azoR, bamC, bcp, bcsB, bcsE, bcsF, bcsG, bcsZ, bdcA, betA, betB, betI, betT, bglJ, bglX, bhsA, bioA, bioB, bioC, bioD, bioF, bluF, bluR, bolA, bssS, btuB, btuD, btuE, btuF, cadA, cadB, cadC, caiA, caiB, caiC, caiD, caiE, caiF, caiT, can, carA, carB, cas1, cas2, cas3, casA, casB, casC, casD, casE, cbl, cbpA, cbpM, ccmA, ccmB, ccmC, ccmD, ccmE, ccmF, ccmG, ccmH, ccp, cdd, cecR, cedA, cfa, chiA, cho, chpB, chpS, cirA, clpA, clpB, clpP, clpX, cmk, cmoM, coaD, cobS, cobT, cobU, codA, codB, copA, corA, cpdB, cpxA, cpxP, cpxR, creA, creB, creC, creD, crp, crr, csgA, csgB, csgC, csgD, csgE, csgF, csgG, csiE, cspA, cspB, cspC, cspD, cspE, csrA, cstA, cusA, cusB, cusC, cusF, cusR, cusS, cutA, cvpA, cyaA, cydA, cydB, cydC, cydD, cynR, cynS, cynT, cynX, cyoA, cyoB, cyoC, cyoD, cyoE, cysA, cysB, cysC, cysD, cysG, cysH, cysI, cysJ, cysK, cysM, cysN, cysP, cysU, cysW, cytR, dacC, dadA, dadX, dam, damX, dapA, dapB, dapD, dapE, dapF, dctA, dctR, dcuA, dcuB, dcuD, dcuR, dcuS, ddlB, deaD, decR, def, degQ, degS, deoA, deoB, deoC, deoD, dgcN, dgcT, dgcZ, dgoA, dgoD, dgoK, dgoR, dgoT, dinB, dinF, dinG, dinI, dinJ, dkgA, dksA, dmsA, dmsB, dmsC, dmsD, dnaA, dnaG, dnaN, dppA, dppB, dppC, dppD, dppF, dps, dsbA, dsbC, dsdA, dsdC, dsdX, dtpA, dusB, dut, dxs, ebgA, ebgC, ecpA, eda, edd, efeU, efp, emrA, emrB, eno, entA, entB, entC, entD, entE, entF, entH, entS, envZ, epd, eptB, era, evgA, evgS, exbB, exbD, exuR, fabA, fabB, fabD, fabF, fabG, fabH, fadA, fadB, fadD, fadI, fadJ, fadR, fau, fbaA, fdnG, fdnH, fdnI, fdx, feaB, feaR, fecI, fecR, feoA, feoB, feoC, fepA, fepB, fepC, fepD, fepE, fepG, fes, fhuA, fhuB, fhuC, fhuD, fhuF, fimA, fimB, fimC, fimD, fimE, fimF, fimG, fimH, fimI, fis, fiu, fixA, fixB, fixC, fkpB, fldB, flgA, flgB, flgC, flgD, flgE, flgF, flgG, flgH, flgI, flgJ, flgM, flgN, flhA, flhB, flhC, flhD, flhE, fliA, fliD, fliE, fliF, fliG, fliH, fliI, fliJ, fliK, fliL, fliM, fliN, fliO, fliP, fliQ, fliR, fliS, fliT, fliZ, flu, fmt, fnr, focA, folA, folE, folK, fpr, frc, frdA, frdB, frdC, frdD, frlA, frlB, frlC, frlD, frlR, frr, frsA, fruA, fruB, fruK, ftnA, ftnB, ftsA, ftsH, ftsI, ftsK, ftsL, ftsQ, ftsW, ftsZ, fucA, fucI, fucK, fucO, fucP, fucR, fucU, fumA, fumB, fumC, fur, gabD, gabP, gabT, gadA, gadB, gadC, gadE, gadX, galE, galK, galM, galP, galR, galS, galT, gapA, garK, garL, garP, garR, gatA, gatB, gatC, gatD, gatY, gatZ, gcd, gcl, gcvA, gcvH, gcvP, gcvR, gcvT, gdhA, gfcA, glcA, glcB, glcC, glcD, glcE, glcF, glcG,*	-0.060

	glgA, glgB, glgC, glgP, glgX, glk, glmS, glmU, glnA, glnB, glnG, glnL, glnQ, glnS, gloA, glpA, glpB, glpC, glpD, glpF, glpK, glpQ, glpT, glpX, glrK, gltA, gltB, gltD, gltF, gltI, gltJ, gltK, gltL, gltX, gluQ, glxK, glxR, glyA, glyQ, glyS, gmk, gnd, gnsA, gntK, gntP, gntR, gntT, gntU, gor, gph, gpmA, gpsA, gpt, grcA, greA, groL, groS, grxB, grxC, grxD, gsk, gspA, gspB, gspC, gspD, gspE, gspF, gspG, gspH, gspJ, gspK, gspL, gspM, gspO, guaA, guaB, gutM, gutQ, gyrA, gyrB, hchA, hdeA, hdeB, hdeD, hemA, hemB, hemF, hemH, hemN, hflC, hflD, hflK, hflX, hfq, hha, hicA, hicB, higA, higB, hipA, hipB, hisA, hisB, hisF, hisH, hisI, hisJ, hisM, hisP, hisQ, hisS, hns, hofB, hofC, hokD, hpf, hpt, hscA, hscB, hslJ, htrE, hupB, hyaA, hyaB, hyaC, hyaD, hyaE, hyaF, hybA, hybB, hybC, hybD, hybE, hybF, hybG, hybO, hyi, hypF, icd, idnK, idnR, ihfB, ileS, ilvA, ilvB, ilvC, ilvD, ilvE, ilvG, ilvH, ilvI, ilvM, ilvN, ilvY, inaA, infA, infB, infC, intQ, intS, iraD, iraM, iraP, iscA, iscR, iscS, iscU, iscX, ispA, ispH, katG, kbaY, kbaZ, kbl, kdpA, kdpB, kdpC, kdsA, kdsB, kdsC, kdsD, lacA, lacI, lacY, lacZ, lamB, lapA, lapB, ldtC, leuA, leuB, leuC, leuD, leuO, lexA, lgt, lipB, livF, livG, livH, livJ, livK, livM, lldD, lldP, lldR, loiP, lolA, lon, lpd, lptA, lptB, lptC, lptD, lpxC, lpxT, lrp, lspA, lsrK, lsrR, lysA, lysC, lysO, lysP, lysR, lysU, lyxK, malE, malF, malG, malI, malK, malM, malP, malQ, malS, malT, malX, malY, manA, manX, manY, manZ, marA, marB, marR, mazE, mazF, mcbA, mdh, mdtE, mdtF, mdtI, mdtJ, melA, melB, melR, menA, mepH, metA, metB, metC, metF, metG, metH, metI, metJ, metK, metL, metN, metQ, mfd, mglA, mglB, mglC, mgtA, mhpA, mhpB, mhpC, mhpD, mhpE, mhpF, mhpR, miaA, mioC, mlc, mlrA, mltF, mngB, mngR, mnmG, mntH, mntP, mntS, modA, modB, modC, moeA, moeB, mpl, mprA, mqsA, mqsR, mraY, mraZ, mreB, mreC, mreD, mrp, msrB, mtlA, mtlD, mtlR, mtn, mtr, mukB, mukE, mukF, murC, murD, murE, murF, murG, murI, murP, murQ, murR, mutL, mzrA, nadB, nadE, nagA, nagB, nagC, nagE, nanA, nanE, nanK, nanT, napA, napB, napC, napD, napF, napG, napH, narG, narH, narI, narJ, narK, narL, narU, narX, ndh, nei, nemA, nemR, nfeF, nfo, nfsA, nfsB, nfuA, nhaA, nhaR, nirB, nirC, nirD, nlpD, nnr, nohA, npr, nrdA, nrdB, nrdD, nrdE, nrdF, nrdG, nrdH, nrdI, nrdR, nrfA, nrfB, nrfC, nrfD, nrfE, nrfF, nrfG, nudB, nuoA, nuoB, nuoC, nuoE, nuoF, nuoG, nuoH, nuoI, nuoJ, nuoK, nuoL, nuoM, nuoN, nupC, nupG, nusA, nusB, obgE, ompA, ompC, ompF, ompN, ompR, ompX, opgG, opgH, osmB, osmC, osmE, osmY, oxc, oxyR, paaA, paaB, paaC, paaD, paaE, paaF, paaG, paaH, paaI, paaJ, paaK, paaX, paaY, pabA, panB, panC, panD, panF, patZ, pck, pcm, pcnB, pdeL, pdhR, pdxJ, pepA, pepD, pepP, pfkA, pflA, pflB, pgi, pgk, pgpA, pgpB, pheA, pheP, phnC, phnD, phnF, phnG, phnH, phnI, phnJ, phnK, phnL, phnM, phnN, phnO, phnP, phoA, phoB, phoE, phoH, phoR, phoU, pitB, pldA, plsX, pncB, pnp, pntA, pntB, polB, poxB, ppdD, ppiA, ppiD, ppsA, pqiA, pqiB, pqiC, preA, preT, prfA, prfC, priB, prlF, prmA, prmC, proP, proS, proV, proW, proX, prpR, prs, psiE, psiF, pspF, pssA, pstA, pstB, pstC, pstS, pta, pth, ptrA, ptsG, ptsH, ptsI, ptsN, purA, purB, purC, purD, purE, purF, purH, purK, purL, purM, purN, purR, purT, putA, putP, pykA, pykF, pyrC, pyrD, pyrF, pyrH, qseB, qseC, rapA, rapZ, rarA, rbfA, rbn, rbsA, rbsB, rbsC, rbsD, rbsK, rbsR, rcnA, rcnB, rcnR, rcsA, rcsB, rcsD, recA, recB, recC, recD, recF, recG, recJ, recN, recO, recQ, recX, relA, relB, relE, rep, rfaD, rfe, rffC, rffG, rffH, rffM, rhaA, rhaB, rhaD, rhaR, rhaS, rhaT, ribA, ribD, ribE, ribF, rihB, rimK, rimM, rimP, rlmE, rluA, rnb, rnc, rnd, rne, rnlA, rnlB, rpe,	

	rpiB, rplB, rplC, rplD, rplI, rplP, rplQ, rplS, rplT, rplU, rplV, rplW, rpmA, rpmC, rpmI, rpoA, rpoD, rpoE, rpoH, rpoN, rpoS, rpoZ, rpsA, rpsB, rpsC, rpsD, rpsF, rpsJ, rpsK, rpsM, rpsO, rpsP, rpsQ, rpsR, rpsS, rpsU, rraA, rraB, rsd, rseA, rseB, rseC, rseD, rsmG, rsmH, rutR, ruvC, safA, sbcC, sbcD, sdhA, sdhB, sdhC, sdhD, sdiA, secB, secG, serA, serC, setA, sfsA, sgbE, sgbH, sgbU, sgrR, sgrT, shiA, sixA, slmA, slp, sodA, sodB, sohB, soxR, soxS, speA, speB, speD, speE, spoT, spy, srkA, srlA, srlB, srlD, srlE, srlR, ssb, ssnA, ssuA, ssuB, ssuC, ssuD, ssuE, stpA, sucA, sucB, sucC, sucD, sufA, sufB, sufC, sufD, sufE, sufS, sulA, sutR, sxy, symE, tatA, tatB, tatC, tatD, tcyJ, tdh, tfaQ, thiB, thiL, thiP, thiQ, thrA, thrB, thrC, thyA, tnaA, tnaB, tomB, tonB, topA, torA, torC, torD, torR, tpiA, tpx, treB, treC, treR, trmD, trmH, trpA, trpB, trpC, trpD, trpE, trpR, trpS, truB, trxA, trxC, tsaE, tsf, tsr, tsx, ttdA, ttdB, ttdT, tufA, tufB, tynA, tyrA, tyrB, tyrP, tyrR, ubiA, ubiC, ubiG, ubiH, ubiI, ubiX, udp, ugpA, ugpB, ugpC, ugpE, ugpQ, uhpT, uidR, ulaA, ulaB, ulaC, ulaD, ulaE, ulaF, umpG, umpH, umuC, umuD, upp, uraA, uspA, uvrA, uvrB, uvrC, uvrD, uvrY, uxaA, uxaB, uxaC, uxuA, uxuB, uxuR, valS, waaA, waaB, waaC, waaF, waaG, waaH, waaJ, waaL, waaO, waaP, waaQ, waaS, waaU, waaY, waaZ, wcaA, wcaB, wecB, wecC, wecE, wza, wzb, wzc, wzxE, wzyE, wzzE, xapA, xapB, xapR, xerC, xerD, xseA, xseB, xylE, yacC, yadS, yadV, yafN, yafO, yafP, yafQ, yagK, yaiA, yajG, yajO, ybbW, ybbY, ybdN, ybdZ, ybeD, ybhF, ybhG, ybhL, ybhR, ybhS, ybiB, ybjC, ybjN, ycaR, yccA, ycdZ, yceI, yceJ, ycgZ, ychA, ychF, ychH, ychO, ychQ, yciB, yciC, yciE, yciH, yciW, yciX, ydeI, ydeJ, ydeM, ydeN, ydeO, ydeP, ydfA, ydfC, ydfE, ydfW, ydfX, ydjM, yeaE, yeaR, yebB, yebC, yebE, yebG, yefM, yegR, yegZ, yehF, yeiB, yfaE, yfdE, yfdV, yfdX, yffB, yfgG, yfiB, yfiR, ygeA, ygeH, ygfB, yhaV, yhbE, yhdT, yhfA, yhgH, yhhY, yhiD, yhjR, yhjX, yiaK, yiaL, yiaM, yiaN, yiaO, yibN, yifL, yigA, yigB, yihI, yihS, yihT, yihU, yihV, yjbE, yjbF, yjbG, yjbH, yjcH, yjjQ, yjjZ, ykgM, ykgO, ykgR, ymgA, ymgC, ymiA, ynfE, ynfF, ynfG, ynfH, yoaE, yoaG, yobF, yoeB, yojI, ypfN, yqhD, yqjA, yrbG, ysgA, ytiC, ytiD, zinT, zntA, znuB, znuC, zwf	

Supplemental Table 4: The top five principal component analysis components of the **A** matrix of PRECISE 2.0 and their contributing factors.

PC Rank	Variance Explained	Top 5 Contributing iModulons (percentage of total weight)
1	17.6%	RpoS (5.72%), FlhDC-2 (4.15%), FliA (3.10%), GadX (2.59%), Translation (1.91%)
2	14.2%	ppGpp (3.55%), RpoS (2.75%), Fnr-2 (2.64%), PurR (2.53%), Fnr-1 (2.38%)
3	8.2%	RpoS (2.06%), FlhDC-2 (1.97%), DksA-related (1.84%), Crp-related (1.82%), Crp-1 (1.80%)
4	6.1%	Anaero-related (2.57%), Fnr-2 (2.53%), Lrp (2.41%), Fnr-1 (2.17%), ArcA (2.15%)

5	5.5%	FlhDC-2 (5.31%), FliA (4.93%), RpoS (4.52%), GadX (4.02%), RhaS (3.31%)

Supplemental Table 5: Genes in various fear vs. greed tradeoff associated iModulons, ordered by iModulon weightings from highest to lowest.

iModulon	Genes
Translation	*rplV, rpsS, rpsC, rplW, rplB, rplP, rplD, rpmC, rpsQ, rplC, rpmD, rpsJ, rplR, rpsE, rplO, rpsR, rplF, rplI, rplA, priB, rpsF, secY, rpsH, rplK, rpsN, fusA, rplM, rplE, rpsG, rpsI, rpsD, rplQ, rpoA, tsf, trmD, rimM, rbfA, rplX, rplS, rpmJ, rpsL, rpsK, truB, rplY, rplN, rpsP, rplL, rpsB, infB, aceF*
ppGpp	*plaP, suhB, rimO, dusB, ydhC, potA, rlmG, yidD, gpt, rluB, queA, fis, rnpA, ydiY, yfhL, ydfO, yegQ, potB, rhlE, ygiQ, rph, rlmC, yegD, rlmF, yciH, pyrF, queD, dusC, pyrD, rpsT, rpsU, mntP, tusA, tsaB, pcnB, ndk, rluC, opgC, yceA, epmA, dbpA, rlmN, yhhQ, fadL, thiI, upp, mltD, mnmA, srmB, ydjX, mnmG, gsk, folK, ycaO, mrcA, rlhA, infA, cmk, cspF, dgcJ, rimI, rplU, miaB, murJ, lysO, trmA, apt, ortT, yggI, rsxB, trmL, recQ, yhbE*
RpoS	*yiaG, ygaM, ycaC, yahO, blc, katE, talA, ycgB, poxB, ybhP, elaB, osmY, otsB, tktB, yodD, ytjA, yciG, yegP, ecnB, patA, yeaG, osmF, ahr, yebV, yjdN, wrbA, yfcG, yccJ, yeaH, ynaL, clsB, yghA, otsA, ggt, msyB, adhP, ybgS, ymgE, yhcO, yeaQ, psiF, yhbO, kbp, yegS, bfr, ybgA, ybdK, fbaB, yehY, ydhS, ybaY, yahK, dgcM, sra, yehW, gabD, yphA, gabT, amyA, yehX, osmC, yohF, yedP, fic, ygdI, dps, yqjE, yqjD, yebF, osmE, yhjG, ydiZ, ybhN, yqjK, yqjC, yjdJ, ydeI, treA, mlrA, aldB, lhgO, tam, yhjY, mcbA, ygiW, yfdC, ybaA, yhfG, curA, gabP, ldtE, sodC, yehE, yciF, yqjG, yjdI, yfiL, ysgA, ydcS, mcbR, yncG, ybjP, yhdW, yohC, ydcK, csiD, ycaP, hchA, chaB, ybeL, yniA, yjgH, yajO, pdeR, yghX, ybiI, yliI, rclA, ldcC, ybhB, ybiO, dkgA, aidB, yjfJ, yjfI*
GadX	*hdeB, hdeD, hdeA, gadA, gadB, gadE, yhiD, gadC, yhiM, rcsB, slp, mdtE, dctR, aidB, mdtF, glsA, ybaT*

Supplemental Table 6: ALEdb experiments used in this paper and their respective ALEdb and PRECISEdb names, if available.

Name on Plots	Short Explanation	Publication	ALEdb Link	PRECISEdb 2.0 Name
2CS KOs	Two component systems were KO'd and evolved	n/a	n/a	Two Component Systems
42C	Heat tolerance evolution	[30]	https://aledb.ucsd.edu/ale/project/1/	42C Evolution
Acid	Acid tolerance	[57]	n/a	Acid

	evolution			
AdnB KO	*AdnB* was KO'd	n/a	n/a	adnB
AntibioticICA	Antibiotic tolerance	n/a	n/a	AntibioticICA
Control	M9 glucose growth with no stressors	[58]	n/a	Control
Cra/Crp KOs	Cra/Crp KO'd	[58]	n/a	Cra/Crp
Crp ARs	KO'd different binding regions of crp	[58]	n/a	Crp ARs
Enzyme Promiscuity	Evolved to enable growth on new substrates	[59]	n/a	Enzyme Promiscuity
Falsely Predicted Essential KOs	KO'd genes that were false positively predicted to be essential	[60]	n/a	False Positives
Fur KOs	*Fur* KO'd	[61]	n/a	Fur
Glucose Evolution	Evolved on M9 glucose with no stressors	[62]	https://aledb.ucsd.edu/ale/project/15/	Glucose Evolution
HOT ALE	Heat tolerance evolution	n/a	n/a	HOT ALE
Misc		n/a	n/a	Misc
Nac/NtrC KOs	Evolved *Nac*/*NtrC* KO's	dissertation - https://escholarship.org/uc/item/0k4709x6	n/a	Nac/NtrC
Naphthoquinone	Evolved obligative naphthoquinone users	[63]	n/a	Naphthoquinone
OmpR KOs	*OmpR* was KO'd and grown	[64]	n/a	OmpR

	under osmotic stress			
Oxidative	Oxidative stress conditions	[65]	n/a	Oxidative
OxyR KO ALE	*OxyR* KO'd and evolved	[8]	n/a	OxyR ALE
RpoB Knock-in	Two *rpoB* SNP mutations introduced	[2]	n/a	RpoB Knock-in
pdhR ALE	*pdhR* was KO'd and evolved	n/a	n/a	pdhR-ALE
pH ALE	Evolved at various pH's	n/a	n/a	pH ALE
Pseudogene Repair	KO'd *entC*, *menF*, and *ubiC* to induce iron import stress and evolved	[66]	n/a	Pseudogene Repair
Respiratory Quinone	*MenF* and *ubiC* deletions and evolutions on glucose	n/a	n/a	Respiratory Quinone
ROS TALE	Evolved cells on various concentrations of paraquat	[38]	https://aledb.ucsd.edu/ale/project/184/	ROS TALE
Substrate - switching Evolution	Evolved on alternating carbon sources	[67]	https://aledb.ucsd.edu/ale/project/42/	Substrate-switching Evolution
SvNS PGI	Various import metabolic genes were KO'd and replaced with exogenous copies, then evolved	Unpublished extension of Sanberg et al. 2020[24]	https://aledb.ucsd.edu/ale/project/51/	SvNS PGI
Various Carbon	Grown on a variety of carbon sources	n/a	n/a	ICA

yTF KO	Unknown function TF’s were KO’d	[68]	n/a	yTF

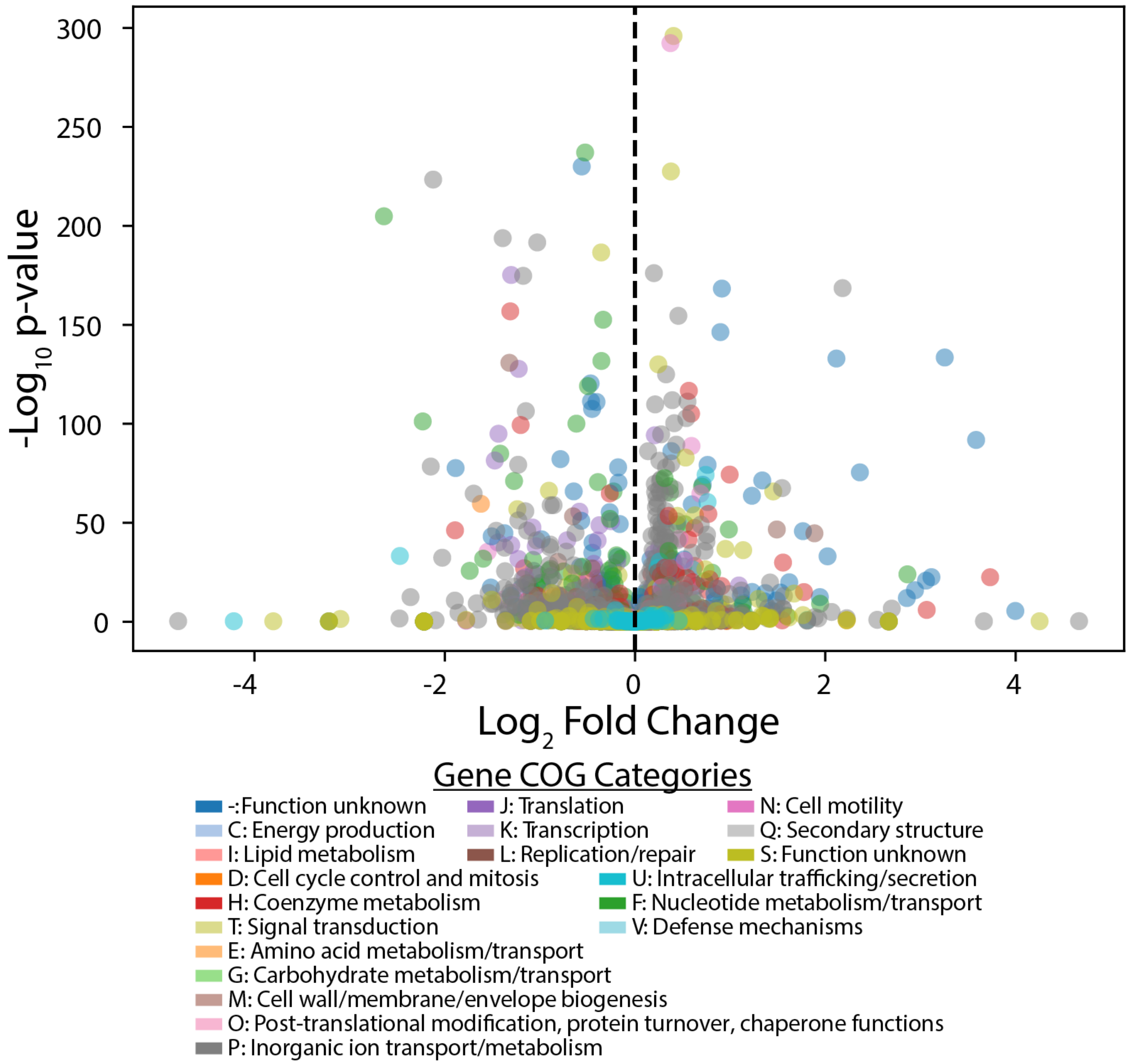

Supplemental Figure 1 - Differential expression genes (DEG) plot between wild-type and the mutated strains colored by COG categories. The median expression value from the mutated strains was used for the mutated strain values. The pairwise single mutant strain compared to the wild-type strain versions of this plot look similar. Interpreting these individual plots is highly difficult and doing so for all these plots together is nearly impossible, thus necessitating the use of iModulon analysis.

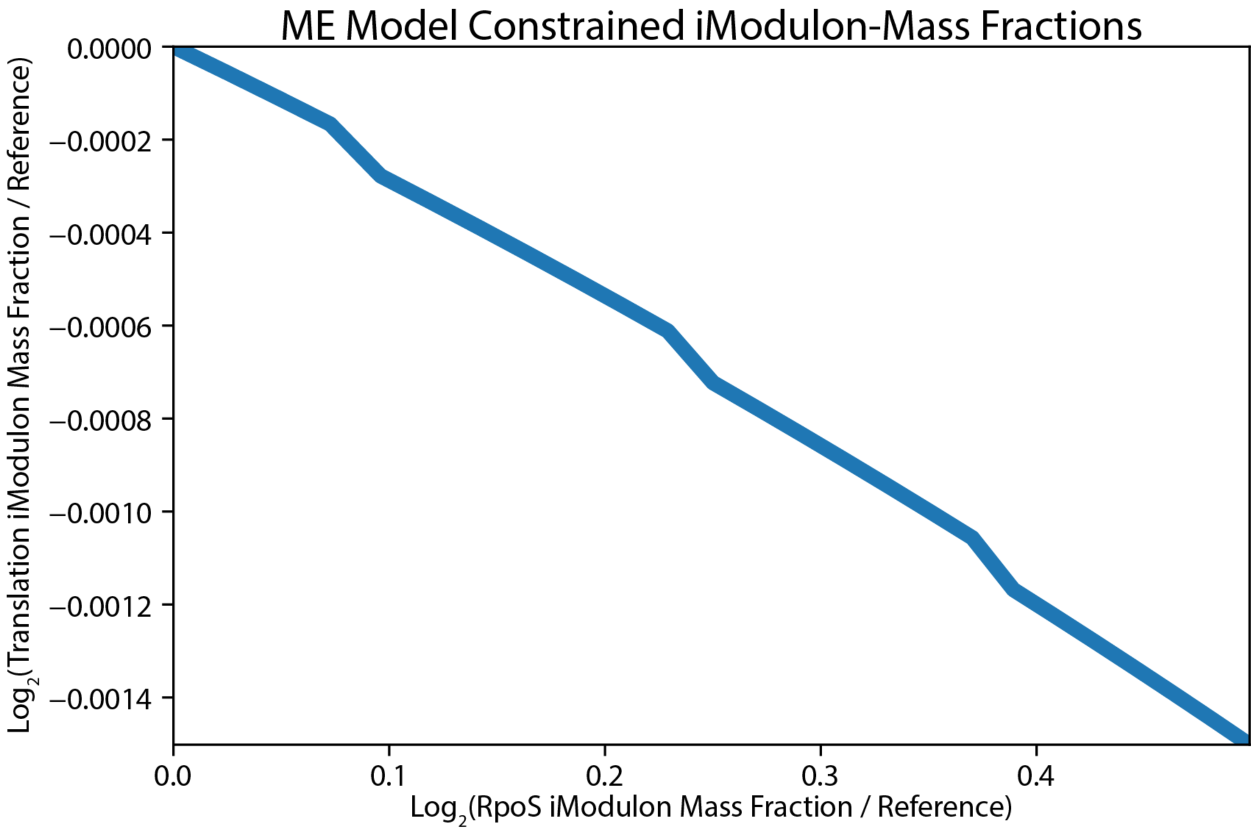

Supplemental Figure 2 - Constrained ME-model simulation predicts fear vs. greed tradeoff. Reactions associated with the Translation iModulon's genes were tightly controlled in a ME model simulation, resulting in a corresponding change in the proteomic mass fraction in both the Translation iModulon and the RpoS iModulon. Growth rates increased nominally (<1%) as RpoS decreased. Note that given the large proteomic fraction allocated to the Translation iModulon compared to that of the RpoS iModulon, its fold-changes are numerically much smaller, but represent a notable proteome reallocation.

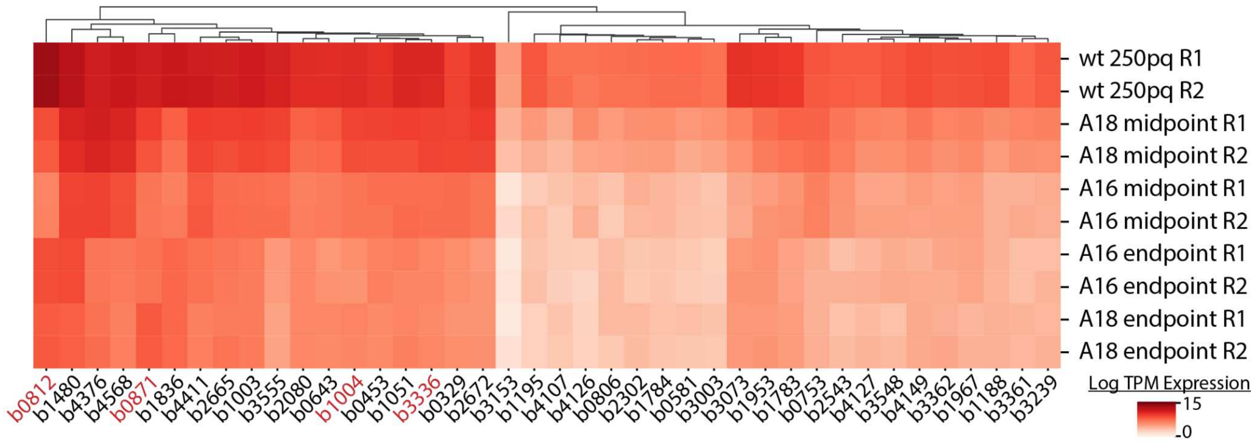

Supplemental Figure 3 - RpoS iModulon's genes downregulating specifically over the evolution. As the cells evolve on 250 μM paraquat, many genes are downregulated from the RpoS iModulon to enable higher growth, but those related to oxidative stress are not (those

highlighted red). Genes listed here are the top 40 most variant within the RpoS iModulon for these strains.

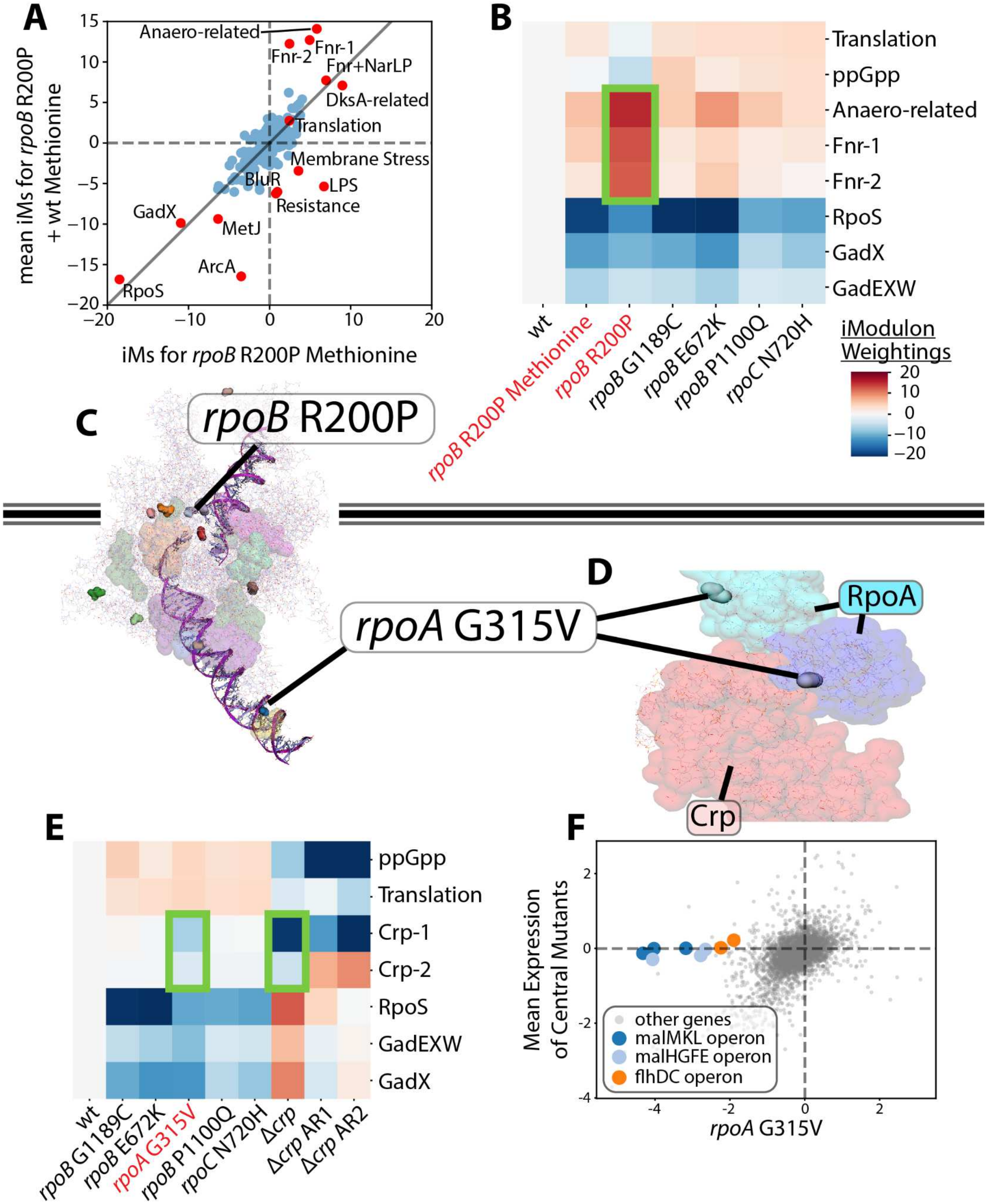

Supplemental Figure 4 - RNAP's ability to adjust to specific stressors. (A) Average iModulon activities of *rpoB* R200P and wild-type methionine compared to iModulon activities of *rpoB* R200P on methionine. **(B)** *RpoB* R200P's specific effect on Fnr and Anaero-related iModulons compared to the most common mutations. **(C)** The location of the two mutations in the whole protein (PDB 6OUL[27]). **(D)** Location of the *rpoA* mutation in relationship to crp (PDB 3N4M[69]). **(E)** *RpoA* G315V's effect on the iModulons, compared to the most common mutations and crp modified strains[37]. (F) Log tpm expression profile of *rpoA* G315V compared to the mean expression of the central mutants.

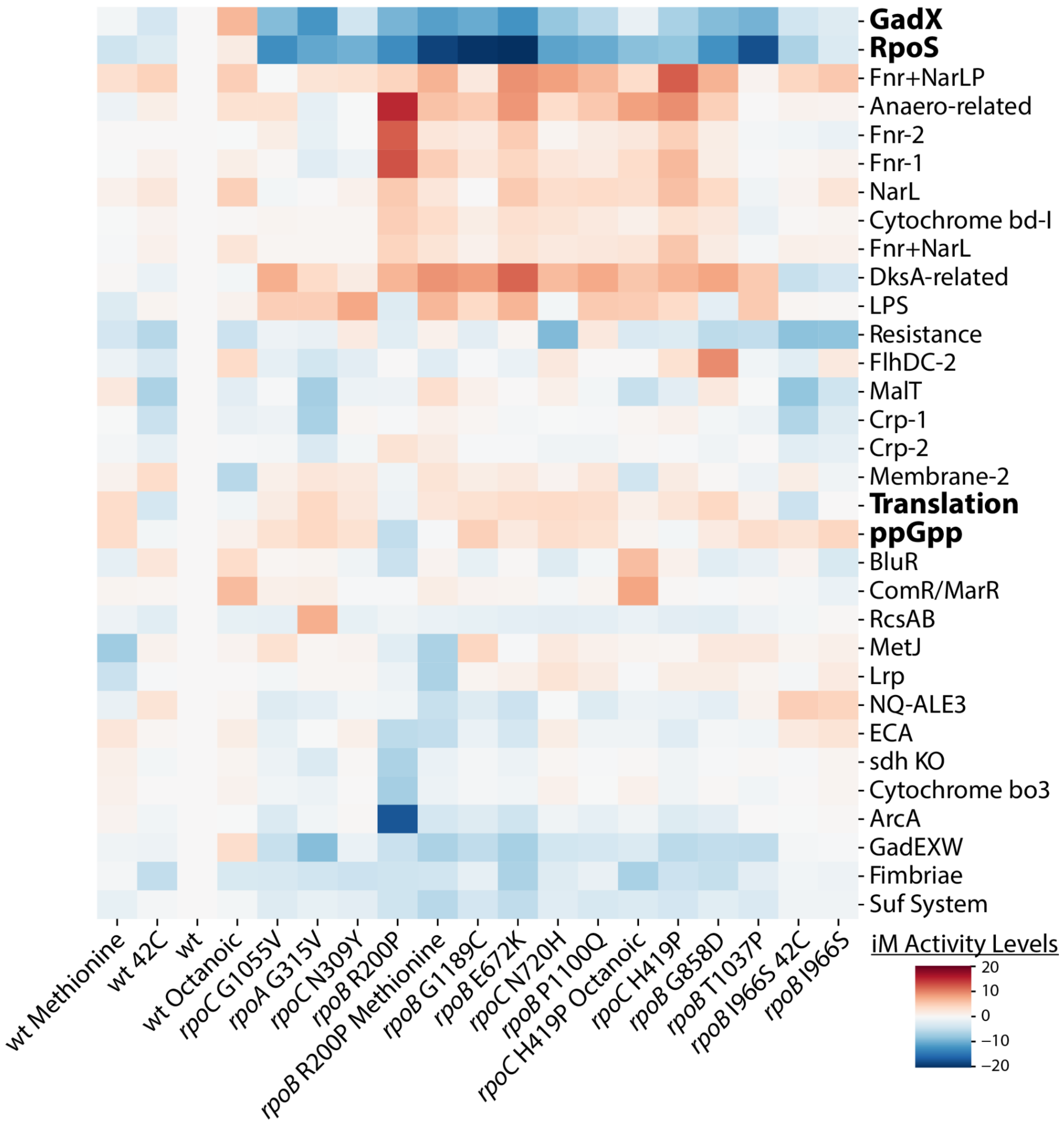

Supplemental Figure 5 - RNAP mutations' largest effects to iModulons. The most differentially activated iModulons for the mutations and reference conditions are shown here. While RpoS is the strongest effect, some other iModulons are modified. The 30 highest variance iModulons for the listed samples are shown along with Crp-1 and Crp-2.
